# Improving the trustworthiness of findings from nutrition evidence syntheses: assessing risk of bias and rating the certainty of evidence

**DOI:** 10.1007/s00394-020-02464-1

**Published:** 2020-12-30

**Authors:** Lukas Schwingshackl, Holger J. Schünemann, Joerg J. Meerpohl

**Affiliations:** 1 Faculty of Medicine, Institute for Evidence in Medicine, Medical Center, University of Freiburg, Freiburg, Germany; 2 Department of Health Research Methods, Evidence and Impact, Department of Medicine, McMaster University, Hamilton, Canada; 3 Cochrane Germany, Cochrane Germany Foundation, Freiburg, Germany

**Keywords:** Trustworthiness, Nutrition evidence, Dietary guidelines, Systematic reviews, Meta-analysis, Risk of bias, GRADE

## Abstract

Suboptimal diet is recognized as a leading modifiable risk factor for non-communicable diseases. Non-randomized studies (NRSs) with patient relevant outcomes provide many insights into diet–disease relationships. Dietary guidelines are based predominantly on findings from systematic reviews of NRSs—mostly prospective observational studies, despite that these have been repeatedly criticized for yielding potentially less trustworthy results than randomized controlled trials (RCTs). It is assumed that these are a result of bias due to prevalent-user designs, inappropriate comparators, residual confounding, and measurement error. In this article, we aim to highlight the importance of applying risk of bias (RoB) assessments in nutritional studies to improve the credibility of evidence of systematic reviews. First, we discuss the importance and challenges of dietary RCTs and NRSs, and provide reasons for potentially less trustworthy results of dietary studies. We describe currently used tools for RoB assessment (Cochrane RoB, and ROBINS-I), describe the importance of rigorous RoB assessment in dietary studies and provide examples that further the understanding of the key issues to overcome in nutrition research. We then illustrate, by comparing the Grading of Recommendations Assessment, Development, and Evaluation (GRADE) approach with current approaches used by United States Department of Agriculture Dietary Guidelines for Americans, and the World Cancer Research Fund, how to establish trust in dietary recommendations. Our overview shows that the GRADE approach provides more transparency about the single domains for grading the certainty of the evidence and the strength of recommendations. Despite not increasing the certainty of evidence itself, we expect that the rigorous application of the Cochrane RoB and the ROBINS-I tools within systematic reviews of both RCTs and NRSs and their integration within the GRADE approach will strengthen the credibility of dietary recommendations.

## Introduction

Non-communicable diseases (NCDs) account for over 70% of total deaths worldwide [1]. According to the Global Burden of Disease studies, suboptimal diet is the leading risk factor for ~ 50% of disabilities from cardiovascular diseases [2]. As in many other areas, systematic reviews (SRs) have been established as the method of choice to synthesize data from primary research studies in the field of nutrition. Hence, the Global Burden of Disease studies, as well as dietary guidelines are based on findings from SRs. Many of these SRs do not exclusively include randomized controlled trials (RCTs), but rely primarily on non-randomized studies (NRSs: in this article the term “NRS” is used exclusively as a synonym for cohort studies), for example prospective cohort studies, because RCTs are not available or considered not applicable [3, 4]. The acceptance of SRs as the main basis for dietary guidelines, Global Burden of Disease studies, and public health nutrition policies constitutes a great opportunity for strengthening the repeatedly criticized trustworthiness (in this article the term “trustworthiness” is used exclusively as a synonym for certainty of evidence [5]) of dietary recommendations [6]. We begin by highlighting the importance and challenges of dietary RCTs and NRSs, and provide potentially less trustworthy results of NRSs, by reporting examples of discordance between findings of RCTs and NRSs. We describe the importance of rigorous risk of bias (RoB) assessment in such studies and provide examples that help understanding the key issues to overcome. We then illustrate, by comparing the Grading of Recommendations Assessment, Development, and Evaluation (GRADE) approach with current approaches (used by United States Department of Agriculture Dietary Guidelines for Americans [7], and the World Cancer Research Fund [8]), how to establish trust in dietary recommendations.

## Why we need more RCTs, and why RCTs are difficult to conduct in the nutrition field?

We need good science to trust dietary advice, ideally unbiased and direct evidence from RCTs to overcome bias. RCTs, if well-designed and -conducted, give robust answers to the research questions they address and are widely encouraged as the ideal methodology for causal inference [9]. However, due to the difficulty of inducing and maintaining dietary changes, randomization to allocate people to alternative diets and to investigate effects of long-term lifestyle behaviors on patient relevant outcomes remains challenging (Table 1). RCT methodologies are accompanied by a number of specific challenges in nutritional research. First, in dietary RCTs, it is often impossible to ensure that participants are unaware of their treatment (except for placebo RCTs of dietary supplements) because people are generally aware of what they are eating. Second, in nutrition research trials, low adherence to a specific dietary regimen is often observed. Third, investigating effects of long-term lifestyle behaviors on patient relevant outcomes is difficult. Well-controlled feeding trials could overcome some of these limitations, since study participants are expected to adhere to strict diet by consuming only food provided by the research kitchen [10]. Moreover, supermarket models have been implemented successfully in RCTs in which the participants receive all groceries free of charge for a period of time, for example, for 6–12 months, in a university supermarket. Bar codes and special computer programs were used to monitor and examine whether the participants followed the right composition of the diets they were allocated to. These intervention models work best for single people, and therefore, generalizability of the results is limited for the general population. Biomarkers of intake have shown that this is a superior method to ensure high compliance and, hence, good validity of the efficacy of the diet intervention [11, 12].

**Table 1:** Strengths and limitations of randomized controlled trials (RCTs) and non-randomized studies (NRSs)

	RCTs	NRSs
Theoretical		
Certainty of the evidence regarding causality	Higher	Lower
Confounding	Unlikely for large RCTs	Adjustment for known and measured confounders possible; residual confounding likely
Levels of exposure	Few; often relatively high differences in intervention groups	Broad range, possibility of stratifying by exposure level
Follow-up time of study	Short or limited	Long
Empirical		
Number of participants	Usually < 1000	Some > 10,000
Representativeness for general population	Often limited	Generally good
Outcome measures	Often risk factor, occasionally morbidity/ mortality	Usually morbidity/ mortality

## Why NRSs provide sometimes potentially less trustworthy results, and how we can identify plausible results?

NRSs, predominately prospective observational studies with patient relevant outcomes (e.g., cardiovascular disease), provide many insights into diet–disease relationships and are the most important source to derive updated Global Burden of Disease studies-reports and dietary guidelines for the primary prevention of NCDs until to date [13]. However, nutrition epidemiology has been repeatedly criticized for providing potentially less trustworthy results. For example, most nutrients not only have been associated with cancer risk but for several of the nutrients there are published reports that show an increased risk in one NRS and a decreased risk in another [14]. In the past, several RCTs comparing dietary interventions with placebo or control interventions have failed to replicate the (presumably protective) associations between dietary factors (e.g., nutrients) and risk for NCDs found in large scale cohort studies [15–19]. For example, RCTs found no evidence for a beneficial effect of fiber intake on CRC risk [20], vitamin E and cardiovascular disease (CVD) [21]. On the contrary, some consistent findings between cohort studies and RCTs have been reported as well (e.g., total fat and coronary heart disease or breast cancer) [22]. Recently, Ioannidis suggested that RCTs should largely replace NRSs in human nutrition research [14] due to the core limitations of NRSs, such as bias due to prevalent-user designs, inappropriate comparators, residual confounding, measurement error, and the fact that small effect sizes are common in nutrition research [23, 24]. Across dietary NRSs, social desirability biases are prevalent: Participants may give perceived “healthy” responses, such as over-reporting fruit and vegetable intake or underestimating fat intake [25], whereas obese patients are more likely to underreport nutritional intake, particularly energy, which can lead to the underestimation of the intake of dietary components assessed [26]. Unfortunately, common tools used to measure dietary adherence in not only NRSs but also RCTs, such as food frequency questionnaires, dietary records, or 24-h dietary recalls, are prone to measurement error [27]. Overall, nutritional research in general poses a number of specific challenges for various empirical approaches.

We postulate that rigorous RoB assessment and the use of the GRADE approach to assess the certainty of the evidence could help to identify plausible results and to address some of the criticism levied at RCTs, NRSs, meta-analyses of such studies, and dietary guidelines, with the aim of overcoming disagreement between classic epidemiologists and interventionalists. This can be done by exploring the nature of potentially less trustworthy results, a process that is often omitted, inconsistently applied across studies, or flawed [28].

## What tools should be used to address risk of bias?

At SR level, the established approach to evaluate the credibility of results from primary studies is RoB assessment. The RoB of a single RCT or RCTs included in a SR should be assessed with a well-established and validated tool, such as the RoB tool by Cochrane [29]. Within the Cochrane RoB tool for RCTs, RoB is assessed for six domains: (i) selection bias, (ii) performance bias, (iii) attrition bias, (iv) detection bias, (v) reporting bias, and (vi) other bias (e.g., carry-over effects in cross-over trials) (Table 2) [29]. In a previous analysis of 50 (18% of them Cochrane Reviews) randomly selected nutrition-specific SRs of RCTs [23], it was shown that 70% used the Cochrane RoB assessment tool [23, 29], 14% reported no RoB assessment, 10% the Jadad Scale [30], and 6% applied their own score. Recently, the RoB 2.0 tool has been published [31]. To this day, dietary adherence has not been included as a specific RoB domain in the Cochrane RoB tool. However in the Cochrane RoB tool 2.0, lack of adherence to a specific dietary intervention will be evaluated within the bias domain assessing deviations from intended interventions [31].

**Table 2:** Comparison of risk of bias domains in RCTs and NRSs, and example of the application of the ROBINS-I tool in a recent meta-analysis investigating the association between adherence to a Mediterranean diet and risk of stroke [51], and the corresponding quality rating by applying the Newcastle–Ottawa Scale

Bias in RCTs	Action that protect against biases	Bias in NRSs	ROBINS-I			Newcastle–Ottawa Scale (max. 9 points)			
(Cochrane RoB tool)			Domains	Rating (*n* = studies, %)		Domains		Rating (*n* = studies, %)	
Bias arising from randomization process	Random sequence generation	Confounding	Bias due to Confounding	Moderate RoB	20/20 (100%)	Comparability of cohorts on the basis of the design or analysis		High quality	20/20 (100%)
	Allocation concealment							Low quality	0/20 (0%)
		Selection	Bias in selection of participants into the study	Low RoB	16/20 (80%)	Representativeness of the exposed cohort		High quality	13/20 (65%)
								Low quality	7/20 (35%)
				Serious RoB	4/20 (20%)	Selection of the non-exposed cohort		High quality	20/20 (100%)
								Low quality	0/20 (0%)
						Demonstration that outcome of interest was not present at start of study		High quality	20/20 (100%)
								Low quality	0/20 (0%)
		Misclassification	Bias in classification of exposure	Low RoB	16/20 (80%)	Ascertainment of exposure		High quality	18/20 (90%)
								Low quality	2/20 (10%)
				Moderate RoB	2/20 (10%)				
				Serious RoB	2/20 (10%)				
Bias due to deviations from intended intervention	Blinding of participants and personnel	Performance	Bias due to deviations from intended exposure	Low RoB	1/20 (5%)	N/A			
				Moderate RoB	19/20 (95%)				
Bias due to missing outcome data	Complete outcome data	Attrition	Bias due to missing outcome data	Low RoB	15/20 (75%)	Adequacy of follow-up of cohorts		High quality	19/20 (95%)
				Moderate RoB	5/20 (25%)			Low quality	1/20 (5%)
Bias in measurement of the outcome	Blinding of outcome assessment	Detection	Bias in measurement of the outcome	Low RoB	19/20 (95%)	Assessment of outcome		High quality	20/20 (100%)
				Serious RoB	1/20 (5%)				
								Low quality	0/20 (0%)
						Was follow-up long enough for outcomes to occur?		High quality	20/20 (100%)
								Low quality	0/20 (0%)
Bias in the selection of the reported results	Avoid selective reporting	Reporting	Bias in the selection of the reported results	Low RoB	14/20 (70%)	N/A			
				Moderate RoB	3/20 (15%)				
				Serious RoB	3/20 (15%)				
			Overall RoB judgement^a^	Low RoB	0/20 (0%)	Overall study quality^b^	High quality (7–9)		20/20 (100%)
				Moderate RoB	13/20 (65%)		Lower quality (0–6)		0/20 (0%)
				Serious RoB	7/20 (35%)				

*N/A* not applicable, *RoB* risk of bias, *ROBINS-I* risk of bias in non-randomized studies of interventions

^**a**^For ROBINS-I overall RoB judgements across studies were based to the most severe of the RoB item-level judgments. Since no single study was judged as low RoB for the domain “confounding”, also in the overall judgement no study was judged with a low RoB

^**b**^For the Newcastle–Ottawa Scale, overall study quality judgements across studies were based on points (0–9). If a single study reached ≥ 7 points, it was defined as high-quality study

Focusing on NRSs, a SR identified 86 tools to assess study quality in NRSs showing high inconsistency in selection/inclusion and weighting of domains across tools [32]. In 50 nutrition-specific SRs of NRSs, it was shown that in 40% of these, no study quality assessment was done [23], 38% used the Newcastle–Ottawa Scale, while the remaining 22% used a variety of other, less well-established tools. When using Newcastle–Ottawa Scale, the most widely applied tool, each study will be judged in relation to eight items (Table 2). However, Stang [33] criticized the NOS for its arbitrary definitions and concluded that this score appeared to be unacceptable for the assessment of study quality, of NRSs. An empirical study has recently shown a fundamental problem when applying the NOS: out of 89 observational nutritional studies, 81 studies (91%) included in 14 meta-analyses were rated as high-quality studies [34]. The threshold to define high quality is apparently so low within NOS that there is no discriminatory effect when applying NOS.

The term “study quality” is often used in this context interchangeably with RoB, but it is important to distinguish between quality and RoB. The term suggests an investigation of the extent to which study authors conducted their research to the highest possible standards. A study may be performed to the highest possible standards yet still have an important RoB. For example, often it is impractical or impossible to blind participants or study personnel to the intervention group. It is inappropriately judgmental to describe all such studies as of “low quality”, but that does not mean they are free of bias resulting from knowledge of intervention status [35]. Moreover, reporting a study (quality) in line with reporting guidelines such as the CONSORT statement (for RCTs) [36] or the STROBE statement [37] is unlikely to have direct implications for risk of bias.

To overcome the problems of the NOS, the risk of bias in non-randomized studies of interventions (ROBINS-I) tool has been developed, and published in 2016 [38] (Table 2). A modified version to assess the RoB in non-randomized studies of exposures (ROBINS-E) is under development, and adaptation of ROBINS for exposure studies is ongoing [39]. For example, Morgan and colleagues published recently a user’s guide on how to apply, interpret, and present the results of ROBINS to assess the RoB in NRSs dealing with effects of exposures (e.g., bisphenol A) on health outcomes (e.g., obesity) [40]. In their user’s guide, the authors applied the draft ROBINS-(exposure) tool successfully, to a variety of study designs including prospective cohort studies and cross-sectional studies [40]; ROBINS-E was also recently used to evaluate RoB in case–control studies [41]. Detailed methods of the application of ROBINS-I and the current development of a RoB instrument for NRSs of exposures have been described in detail by Morgan and colleagues [42]. For example, domains 3 (“bias in classification of interventions”) and 4 (“bias due to departures from intended interventions”) of ROBINS-I have been changed to “bias in classification of exposures” and “bias due to departures from exposures” [42] (Table 2). The COSMOS-E reporting guideline (Conducting Systematic Reviews and Meta-Analyses of Observational Studies of Etiology) has recently been published; COSMOS-E also recommends the use of the ROBINS tool to evaluate RoB in observational studies, and the GRADE approach to rate the certainty of evidence [43]. GRADE is not recommending a specific tool to assess RoB, however, because the tools have different advantages and disadvantages that influence the choice. As long as RoB is assessed across studies, any validated and appropriate tool can be used.

## Examples of critical risk of bias in certain domains in a dietary Cochrane Review

To exemplify the usage and judgements of Cochrane RoB domains for dietary RCTs, we chose a highly cited Cochrane Review on Mediterranean diet (MedDiet) and prevention of CVD, which included 49 papers. In this Cochrane Review, the authors took into account the difficulties of blinding participants (although, double blinded designs are not always ideal for providing a reliable answer to the trial’s research question [44]) in dietary interventions and rated this as unclear rather than high RoB [45]. The procedure of the author’s shows that especially for the RoB assessment of dietary RCTs, a puristic approach to judge RoB is not always sensible. Dietary adherence is probably the most important limitation of dietary RCTs, mainly in long-term RCTs [46]. Not only has dietary adherence not been assessed as a RoB item to date, but also many SRs do not even investigate dietary adherence at all, like our exemplary chosen Cochrane Review on MedDiet and CVD [45].

Difficulty of attrition (in free living populations, a 40–50% dropout rate is fairly common [47]) is mainly observed in longer-term dietary RCTs [48]. In the Cochrane Review on the MedDiet and CVD, only two out of 30 RCTs conducted intention-to-treat analyses, and only 11 RCTs were rated with a low RoB for attrition bias [45].

## Why is risk of bias so important to evaluate the credibility of study results?

Sensitivity analyses including only low RoB- or excluding high-RoB RCTs are an important means to explore the impact of bias on pooled results in a meta-analysis. A methodology study evaluating 59 SRs showed that only 50% of these SRs conducted sensitivity analyses for low RoB studies [49]. In some circumstances, when conducting sensitivity analyses excluding trials with a high RoB, significant summary estimates become statistically non-significant or vice versa. For example, in a large Cochrane Review investigating the effects of antioxidant supplements for prevention of mortality, risk increasing effects for beta-carotene and vitamin E were only observed in the sensitivity analyses for low RoB RCTs, whereas the primary analysis showed no effects [50]. Because the ROBINS-I tool has only recently been published, it lacks application in nutrition-specific meta-analyses. A recent SR of NRSs investigating the relationship between adherence to the Mediterranean Diet and risk of stroke applied the ROBINS-I tool [51]. Out of 20 included studies, no NRS was rated with a low RoB, 13 NRSs (65%) were rated as moderate RoB, and seven NRSs (35%) as serious RoB (Table 2). On the contrary, the application of the NOS in those studies resulted in a high-quality (low RoB) judgement for all 20 NRSs (Table 2). Rigorous application of the Cochrane RoB tool for RCTs and the ROBINS-I tool for NRSs, would improve evaluation of validity, transparency, interpretation and conclusions of a single dietary RCT or RCTs included in a SR.

## Why can rating the certainty of evidence improve the trustworthiness of findings?

RoB assessment is a fundamental part of the GRADE approach. The GRADE working group [52] has developed a common and transparent approach for grading the certainty of evidence and strength of recommendations based on a body of evidence (e.g., SR of RCTs). GRADE is also used in the field of nutrition research [53]. The GRADE approach classifies bodies of RCTs as initially starting at high certainty and bodies of NRSs at initially starting at low certainty [54]. In 2016, the NutriGrade scoring system by Schwingshackl and colleagues was published [23]. The main proposed adaptation of GRADE was a modified initial classification of a body of evidence of RCTs and cohort studies. NutriGrade, and its proposed adaptations was discussed extensively in the scientific community [55, 56]. Afterwards, the GRADE working group has acknowledged limitations that in certain research fields (e.g., nutrition research), RCTs on patient relevant outcomes (e.g., cardiovascular disease) are sparse or not feasible, and that the application of the current GRADE approach to classify study designs might be limited. It has been pointed out that using the GRADE approach, in particular in relation to RoB assessment, is challenging and may lead to excessive downgrading. For example, GRADE users may inappropriately double count the risk of confounding and selection bias by downgrading the initial body of evidence to low, followed by further downgrading due to unknown confounders [23, 56–58]. Therefore, guidance on how to assess the certainty of evidence within GRADE when ROBINS-I is being used was published in 2018. RoB instruments, such as ROBINS that allow for the comparison of a body of evidence from NRSs to RCTs eliminate the GRADE requirement for starting an assessment of a body of evidence as “high” or “low” certainty based on study design (Table 3) [57]. This will lead to a better comparison of evidence from RCTs and NRSs because they are placed on a common metric for RoB. Due to its enhanced development we are now suggesting the GRADE approach to rate the certainty of the evidence.

**Table 3:** Main methodological differences between the GRADE approach [54], and approaches taken by the 3rd World Cancer Research Fund/ American Institute for Cancer Research Expert report [8], and the USDA Dietary Guidelines for Americans 2015–2020 [7] to rate the certainty of evidence

Macro-level	Micro-level	GRADE		3rd WCRF/AICR Report^a^		USDA DGA 2015–2020^b^	
		Y/N/U	Explanation	Y/N/U	Explanation	Y/N/U	Explanation
A priori assumption	Body of evidence (RCTs and cohort studies) begins as high certainty	N	Using NOS	Y	N/A	Y	N/A
		Y	Using ROBINS-I				
Certainty criteria	Risk of bias (aka study quality)	Y	Cochrane RoB tool for RCTs; NOS or ROBINS-I for NRS	U	No tool; some criteria reported: confounding, measurement error and selection bias (but assessment unclear)	Y	Tool adapted from Cochrane: selection bias, performance bias, detection bias, attrition bias
	Inconsistency	Y	Similarity of point estimates, extent of overlap of 95% CI, statistical criteria including tests of heterogeneity, and I^2^	Y	I^2^	U	Description vague: e.g., consistent in direction and size of effect or degree of association and statistical significance with very minor exceptions
	Indirectness	Y	According to PICO criteria	N	N/A	Y	According to PICO criteria
	Imprecision	Y	Examination of 95% CI, OIS	N	N/A	U	Large sample size
	Publication bias	Y	Funnel plot	N	N/A	N	N/A
	Large effect	Y	RR: > 2 or < 0.5 (large effect) RR: > 5 or < 0.2 (very large effect)	Y	RR: > 2 (large effect)	U	Description vague: e.g., clinically meaningful effect size
	Dose–response	Y	Linear, non-linear	Y	Linear, non-linear	N	N/A
	Plausible residual confounding	Y	Plausible confounders would decrease an effect, or would create a spurious effect when results suggest no effect	N	N/A	N	N/A
Transparent Summary	Summary of Findings Table	Y	Certainty rating (and criteria) for each outcome and the estimate of effect	N	N/A	N	N/A
Overall rating	Certainty of evidence	High, Moderate, Low, Very low		Convincing, Probable, Limited-suggestive, Limited-no conclusion, Substantial effect on risk unlikely		Strong, Moderate, Limited, Grade not assignable	

*95% CI* 95% confidence interval, *I*^*2*^ inconsistency, *N* no, *N/A* not applicable, *NOS* Newcastle Ottawa Scale, *OIS* optimal information size, *PICO* population, intervention, comparison, outcome, *ROBINS-I* risk of bias in non-randomized studies of interventions, *RR* risk ratio, *U* unclear; Y: yes

^a^World Cancer Research Fund/American Institute for Cancer Research report is the most comprehensive global research project on diet, and physical activity and cancer risk or survival [8]

^b^The United States Department of Agriculture’s Dietary Guidelines for Americans 2015–2020 are the most comprehensive guidance for healthy eating in the US and worldwide [7]

The GRADE domains: RoB, inconsistency, indirectness, imprecision, publication bias, large effect, dose–response, and direction of plausible residual confounding are taken into account to arrive at the certainty of the evidence for a given outcome across studies (Table 3). Overall, GRADE specifies four levels of certainty of evidence: high, moderate, low, and very low. For example, high certainty of evidence is defined as: “High certainty that a true effect lies on one side of a specified threshold or within a chosen range” [5]. Guideline authors then consider the direction and strength of recommendation (strong vs. conditional) [59] based on overall certainty of evidence across outcomes and in light of various other criteria including values or importance of the outcomes, resource use, equity, acceptability and feasibility [60]. Although only few nutrition-specific SRs have evaluated the certainty of evidence so far, the GRADE approach is now being applied increasingly in nutrition research [23, 58].

## What are current approaches for making dietary recommendations?

Table 3 highlights the main methodological differences in rating the certainty of evidence by comparing the GRADE approach with the approaches by the WCRF [8] and the USDA Dietary Guidelines for Americans 2015–2020 [7]. Overall, the GRADE approach provides more transparency about the single domains.

Separating certainty of the evidence assessment from confidence in a recommendation and decision will bring clarity to the field, lead to better research and could overcome the incommunicado between different stakeholders. Table 4 highlights the Evidence to Decision (EtDs) framework which aims to use evidence in a systematic and transparent way to inform decisions established by the GRADE working group [61]. Guidelines approaches neither by the WCRF nor the USDA have integrated patient and community values and preferences, nor applied strict safeguards against conflicts of interest. However, several components of the EtDs framework have been addressed to be implemented in future UDSA dietary guidelines. In the future, developers of recommendations should take a population perspective for general public health nutrition recommendations, and may consider also biological plausibility or sustainability, which are highly relevant for dietary recommendations [62, 63]. Biological plausibility is a domain which is considered by the WCRF to rate the certainty of evidence [8], whereas GRADE does not consider the issue of biological plausibility as domain of certainty. GRADE argues that biological plausibility is considered in three ways: (1) during question formulation; (2) in the evaluation of other, indirect evidence (e.g., similar nutrient or population); and (3) how directly the intervention affects a surrogate outcome [64].

**Table 4:** Main methodological differences between the GRADE Evidence to Decisions framework [60], and approaches taken by the 3rd World Cancer Research Fund/ American Institute for Cancer Research Expert report [8], and the USDA Dietary Guidelines for Americans 2015–2020 [7]

Macro-level	Micro-level	GRADE		3rd WCRF/AICR Report^a^		USDA DGA 2015–2020^b^	
		Y/N/U	Explanation	Y/N/U	Explanation	Y/N/U	Explanation
Conflict of interest from committee members	Intellectual and financial conflicts	Y	Should be reported	N	Not reported	U	Per Federal Advisory Committee Act rules, Advisory Committee members were thoroughly vetted for conflicts of interest before they were appointed to their positions and were required to submit a financial disclosure form annually
EtDs framework	Problem	Y	Problem priority definition?	U	N/A	U	N/A
	Benefit and harms	Y	How substantial are beneficial/harmful effects?	U	N/A	N	N/A
	Certainty of evidence	Y	see Table 2	Y	see Table 2	Y	see Table 2
	Values	Y	How much people value the main outcomes?	N	N/A	N	N/A
	Balance of effects	Y	Balance between desirable and undesirable effects?	N	Panel has sometimes not made recommendations despite strong evidence; because of potentially adverse effects (dairy and prostate cancer) on one cancer despite evidence of protection for another (e.g., dairy and colorectal cancer)	N	N/A
	Resources required	Y	How large are the costs?	N	N/A	N	N/A
	Cost effectiveness	Y	Cost effectiveness of the intervention?	N	N/A	N	N/A
	Equity	Y	Impact on health equity?	N	N/A	N	N/A
	Acceptability	Y	Option acceptable to key stakeholders?	N	N/A	N	N/A
	Feasibility	Y	Implementation feasible?	U	Sometimes not feasible	N	N/A
Recommendation	Strength of recommendation	Y	Strong, weak (conditional, discretional, or qualified)	Y	Recommendations were made only when the CUP Panels judged the evidence sufficiently strong (when exposure was convincingly/probably or causally linked to cancer risk)	U	The grades used for conclusion statements also fall into one of four categories (as the certainty of evidence evaluation): strong, moderate, limited, and grade not assignable

*CUP* continuous update project, *EtDs* Evidence to Decisions, *N* no, *N/A* not applicable, *U* unclear, *Y* yes

^a^World Cancer Research Fund/American Institute for Cancer Research report is the most comprehensive global research project on diet, and physical activity and cancer risk or survival [8]

^b^The United States Department of Agriculture’s Dietary Guidelines for Americans 2015–2020 are the most comprehensive guidance for healthy eating in the US and worldwide [7]

## Conclusion

Despite not increasing the certainty of evidence itself, we expect that the rigorous application of the Cochrane RoB and the ROBINS-I tools within SRs of both RCTs and NRSs and their integration within the GRADE approach will strengthen the credibility of dietary recommendations.

## Financial support

This research received no specific grant from any funding agency, commercial or not-for-profit sectors.

## Abbreviations

GRADE: Grading of Recommendations Assessment, Development, and Evaluation
NRSs: Non-randomized studies
RCTs: Randomized controlled trials
RoB: Risk of bias
ROBINS-I/E: Risk of bias in non-randomized studies of interventions/exposures
SRs: Systematic reviews
WCRF: World Cancer research Fund
